# Evaluation of the Copy Number Variants and Single-Nucleotide Polymorphisms of *ABCA3* in Newborns with Respiratory Distress Syndrome—A Pilot Study

**DOI:** 10.3390/medicina60030419

**Published:** 2024-02-29

**Authors:** Mădălina Anciuc-Crauciuc, Manuela Camelia Cucerea, George-Andrei Crauciuc, Florin Tripon, Claudia Violeta Bănescu

**Affiliations:** 1 Genetics Department, George Emil Palade University of Medicine, Pharmacy, Science, and Technology, 540142 Târgu Mureș, Romania; madalina.anciuc@umfst.ro (M.A.-C.); florin.tripon@umfst.ro (F.T.); 2 Neonatology Department, George Emil Palade University of Medicine, Pharmacy, Science, and Technology, 540142 Târgu Mureș, Romania; manuela.cucerea@umfst.ro; 3 Genetics Laboratory, Center for Advanced Medical and Pharmaceutical Research, George Emil Palade, University of Medicine, Pharmacy, Science, and Technology of Târgu Mureș, Gheorghe Marinescu 38, 540139 Târgu Mureș, Romania

**Keywords:** *ABCA3* gene, respiratory distress syndrome, newborns

## Abstract

*Background and Objectives*: Respiratory distress syndrome (RDS) in preterm infants commonly occurs due to the immaturity-related deficiency of pulmonary surfactant. Beyond prematurity, various environmental and genetic factors can influence the onset and progression of RDS. This study aimed to analyze three single-nucleotide polymorphisms (SNPs) of the *ABCA3* gene to assess the *ABCA3* gene as a candidate gene for susceptibility to RDS and overall survival in newborns and to evaluate the utility of MLPA in RDS neonatal patients. *Materials and Methods*: Three SNPs were chosen and genotyped in a cohort of 304 newborns. Data analysis and statistical tests were employed to examine allele frequencies, haplotypes, and measures of pairwise linkage disequilibrium. *Results*: There was no observed haplotype association with SNPs rs13332514 (c.1059G>A) and rs170447 (c.1741+33T>C) among newborns, both with and without RDS (*p* > 0.05). The minor C allele frequency of the *ABCA3* rs323043 (c.1755G>C) SNP showed a significant increase in preterm infants with RDS. MLPA results indicated that the predominant findings were normal, revealing no CNVs in the genes *ABCA3* and *SFTPC* that were investigated in our patients. *Conclusions*: The presence of the variant C allele in the rs323043 (c.1755G>C) SNP may be a risk factor for RDS in premature newborns.

## 1. Introduction

Recent progress in high-throughput sequencing technologies has enabled the identification of extensive genomic data. These data are utilized to create comprehensive catalogs of genetic variations in both disease-affected and healthy individuals [1]. These genetic variants form the foundation of distinct characteristics linked to susceptibility to particular diseases and/or drug responses. The most frequent genetic variants in the human genome are single-nucleotide polymorphisms (SNPs), which occur with a frequency exceeding 1% in the population [2].

The higher heritability of these traits implies the presence of additional genetic influences beyond what common SNPs can account for [3]. These influences may be attributed to structural variants (SVs), alterations in the genome affecting at least 50 bp, and significant and rare pathogenic variants. SVs include variations like deletions, duplications, insertions, inversions, or chromosomal translocations. It is noteworthy that SVs can manifest as insertions and deletions (indels), and conversely, indels represent a subset of SVs. While less common in the human genome compared to single-nucleotide variations (SNVs) or small insertions or deletions (INDELs; <1 kb), structural variants (SVs) encompass a greater number of base pairs and may lead to a more significant pathological impact [4]. In 2015, the consortium of the 1000 Genomes project discovered that a typical genome encompasses 2100 to 2500 SVs, covering 20 million bases [5]. Copy number variants (CNVs; ≥1 kb) are a subtype of SVs that specifically impact the number of copies of a particular genomic region carried by an individual. The biological functions of CNVs span from apparently negligible impact on the typical variability of physiological traits to influencing morphological diversity, altering metabolic states, impacting susceptibility to infectious diseases, and playing a role in host–microbiome interactions [6,7,8,9]. CNVs can significantly contribute to both common and rare genetic disorders/syndromes [10]. Consequently, they possess considerable potential to influence the diversity of human populations and contribute to micro- and macro-evolutionary processes [11,12,13].

The techniques used to identify SVs diverge from those utilized for detecting SNVs and INDELs. Specifically, contemporary sequencing methods relying on short reads, proficient in identifying SNVs and INDELs, may encounter limitations in effectively detecting and characterizing SVs. Multiplex ligation-dependent probe amplification (MLPA), a targeted analytical approach utilizing multiplex PCR, has found widespread application in identifying SVs in various genetic disorders. While MLPA excels at detecting the gain or loss in genetic material, typically exons, it does have limitations in precisely determining the length of SVs and does not achieve nucleotide resolution [14].

Gene expression studies have highlighted the importance of surfactant proteins (SPs) A, B, C, and D, along with other proteins such as A3 member of the ATP binding cassette family (ABCA3) and thyroid transcription factor 1 (TTF-1), in functional surfactant production. These proteins, expressed permanently with increased peak expression in later gestational stages, are critical for lung surfactant metabolism. Neonatal respiratory distress syndrome may result from variants in the *ABCA3* gene or genes encoding surfactant proteins (SFTPA, SFTPB, SFTPC, SFTPD) [15,16,17,18,19,20,21,22]. These genes are crucial for encoding essential surfactant components and facilitating the organized assembly and packaging of surfactant phospholipids within lamellar bodies. Additionally, they play a significant role in facilitating the uptake of surfactant phospholipids onto the alveolar surface [23]. While a genotype–phenotype correlation has not been universally demonstrated for all surfactant genes, various genetic factors, including interacting genes, variations in regulatory regions, and environmental influences, contribute to expression variability [24]. Mutations in the *ABCA3* gene, via autosomal recessive inheritance, lead to fatal respiratory failure in term infants. Different common variants in *ABCA3* have been previously described at the population level to establish correlations between genotype and phenotype [25]. Variants in the *SFTPC* gene exhibit an autosomal dominant inheritance pattern, varying in expression levels and penetration. Furthermore, approximately 50% are de novo and 50% are inherited [17].

Among single-nucleotide variants (SNVs), various effects are observed, and following their clinical significance, the classification of variants of *ABCA3* in the genome Aggregation Database (gnomAD) v4.0 are reported as follows: pathogenic (0.15%), likely pathogenic (0.21%), conflicting interpretation (0.94%), uncertain significance (3.79%), likely benign (1.64%), benign (0.32%), and unknown (92.62%). Considering the high percentage of unknown variants that could be associated with RDS, studies on different populations are needed to establish their clinical significance.

Previously conducted research performed both on preterm and full-term newborns has suggested a potential impact of *ABCA3* rs170447 (c.1741+33T>C), rs323043 (c.1755G>C), and rs13332514 (c.1059C>T) variants on the risk and/or prognosis of RDS [26,27]. Despite in silico predictions indicating preserved protein function, conflicting evidence exists regarding the RDS risk in case–control studies [26,28,29].

Given the current understanding and the inconsistencies in findings, our study aimed to explore the hypothesis proposing a correlation between *ABCA3* rs170447 (c.1741+33T>C), rs323043 (c.1755G>C), and rs13332514 (c.1059G>A) variants and the likelihood of neonatal RDS development, as well as overall survival within the Romanian population. Furthermore, our study investigated the utility of MLPA analysis in neonatal patients with RDS.

## 2. Materials and Methods

The ethics committees of George Emil Palade University of Medicine, Pharmacy, Science and Technology of Targu Mures and Emergency Clinical County Hospital Targu Mures previously approved the study protocol (renewed approval no. 23520/9 October 2023). The subjects were included in the study only after obtaining the parents’ signed consent, and the ethical principles in research activities were applied according to the Declaration of Helsinki.

### 2.1. Study Population

The study enrolled 290 preterm infants, white European descendants, born <36 weeks of gestation, and hospitalized at Targu Mures Emergency Clinical County Hospital Maternity, a tertiary hospital. The patients were categorized based on the presence of RDS, with 214 infants in the control group showing no signs of RDS. The diagnosis of RDS was established based on specific criteria, including the onset of respiratory distress within the initial hours of life, a requirement for continuous positive airway pressure (CPAP) or mechanical ventilation within the first 48 h of life, and the observation of characteristic radiological findings in chest X-rays. Confirmation of the diagnosis involved identifying symptoms such as tachypnea (>60 breaths/min), chest retractions, nasal flaring, grunting, and the necessity to maintain oxygen saturation at ≥86% with FiO_2_ ≥ 0.40. Additionally, the diagnosis was supported by chest radiograph results displaying at least Grade 2 RDS findings and a Silverman–Andersen Score above 6 [30,31].

### 2.2. Molecular Analysis

DNA extraction was performed from peripheral blood using NucleoSpin Blood QuickPure (Macherey-Nagel GmbH & Co, KG, Valencienner Str. 11, Düren, Germany) according to the manufacturer’s protocol. The DNA concentration and absorbance were determined by a spectrophotometric technique using Eppendorf BioSpectrometer (Eppendorf AG, Barkhausenweg 1, Hamburg, Germany). The absorbance ratio at 260 nm to 280 nm serves as an additional indicator of the purity of DNA. A minimal concentration of 10 ng/uL was mandatory for each sample; otherwise, it was necessary to repeat DNA extraction. Furthermore, the concentration and the absorbance of DNA were a significant criterium for the repetition of DNA extraction, with the MLPA technique being more sensitive to impurities than other PCR assay techniques. The reference DNA samples were obtained from healthy subjects who were previously tested and were negative for CNVs.

The MLPA analysis was performed using SALSA MLPA Probemix P314-A1 ABCA3-SFTPC (MRC-Holland, Willem Schoutenstraat 1, 1057 DL, Amsterdam, The Netherlands), and the protocol was performed according to the manufacturer. The probe mix contains 28 MLPA probes for the *ABCA3* gene, analyzing all exons except exons 6, 12, 13, 31, and 33. For *SFTPC*, the kit used contains six probes, covering all the exons of this gene. The MLPA-PCR products were analyzed using GeneScan™ 600 LIZ™ dye Size Standard v2. (ThermoFisher Scientific, 168 Third Avenue, Waltham, MA, USA) and the Applied Biosystems 3500xL Genetic Analyzer (Applied Biosystem, 850 Lincoln Centre Dr., Foster City, CA, USA), with a 50 cm capillary and POP-7 polymer. Data analysis was performed using Coffalyser.Net v.220513.1739 software and the specific MLPA Coffalyser sheet, according to the lot number. For data normalization, we used three reference samples in each MLPA analysis. These samples were obtained from similar tissue (blood). The interpretation of the results for each probe followed the manufacturer’s criteria, with inclusion in the study granted to those exhibiting a standard deviation lower than 0.10. For the reference samples, acceptance criteria included a dosage coefficient ranging from 0.80 to 1.20.

In the second step, three SNPs were selected: rs170447 (c.1741+33T>C), rs323043 (c.1755G>C), and rs13332514 (c.1059G>A) in the *ABCA3* gene [26,27]. We performed a case–control study using real-time polymerase chain reaction (PCR) and TaqMan assays (ThermoFisher Scientific, 168 Third Avenue, Waltham, MA, USA) C___9581071_10, C___3156784_10, and C__25970779_10. Genotyping of *ABCA3* rs170447 (c.1741+33T>C), rs323043 (c.1755G>C), and rs13332514 (c.1059G>A) polymorphisms was performed using the 7500 Fast Dx real-time PCR system (Applied Biosystem, 850 Lincoln Centre Dr., Foster City, CA, USA). The description and annotation of the *ABCA3* variants were obtained from the dbSNP database and the Ensemble genome browser Variant Effect Prediction tool, respectively [32,33].

### 2.3. Data Analysis

#### 2.3.1. Descriptive Analysis

Demographic and clinical information was conveyed using descriptive statistics, expressed as means ± standard deviations for numerical data or percentages and absolute frequencies for categorical data.

#### 2.3.2. Inferential Analysis

The statistical analysis in the current study was conducted using the Statistical Package for Social Sciences (SPSS, version 20, Chicago, IL, USA). Associations between genotype distribution and categorical variables were assessed using contingency tables and the chi-square test, with Yates correction when appropriate. Fisher’s exact test was applied when the theoretical value was less than 5 in more than one table cell. The *t*-test was applied to data that exhibited a normal distribution. To evaluate the probability or susceptibility to RDS associated with specific polymorphisms, odds ratios (ORs) were calculated. The deviation of allelic frequencies from Hardy–Weinberg equilibrium was determined using a chi-square test. Each polymorphism’s potential as a predictor for RDS was analyzed through simple binary logistic regression. In the logistic regression model, we included the gestational age and gender to assess the potential impact of the investigated SNPs. The significance threshold was set at α = 0.05, with measures below this threshold considered statistically significant.

The analysis of haplotypes for the studied RDS gene polymorphisms was conducted utilizing Haplotype Analysis software version 1.05, available from Georg-August-Universität Göttingen.

## 3. Results

### 3.1. Clinical Characteristics of the Patients

A total of 290 DNA samples were genotyped, including 76 from the infants diagnosed with RDS according to European Consensus Guidelines [34] and 214 samples from the control group displaying no signs of RDS. The RDS group exhibited a lower average gestational age (31 ± 3.72 vs. 32 ± 2.80) and birth weight (1473.10 ± 504.51 vs. (1620.20 ± 310.5) in comparison to the control group. The gender distribution favored males in both groups, 65.5% in the patients’ group and 62.6% in the control group. Tocolytic therapy was administered to inhibit acute preterm labor in 18.4% (14 cases) within the RDS group. Surfactant was not administered to any infants before the RDS diagnosis. In the RDS group, 69 of the newborns recovered and were discharged. Infants who did not recover from RDS and progressed to develop chronic lung disease necessitated supplemental oxygen or ventilation for diffuse lung disease at the postnatal age of 28 days, also referred to as mild bronchopulmonary dysplasia (BPD). Seven newborns died despite intensive care and surfactant replacement therapy; all of them received surfactant more than once, given the severe form of RDS and inadequate response to treatment. All seven patients had unremarkable workup findings (including serial infection markers, echocardiography, and blood and sputum pathogen testing). Complete clinical and demographic data for the study samples are provided in Table 1.

### 3.2. CNV Analysis in RDS Patients

**Figure 1:**
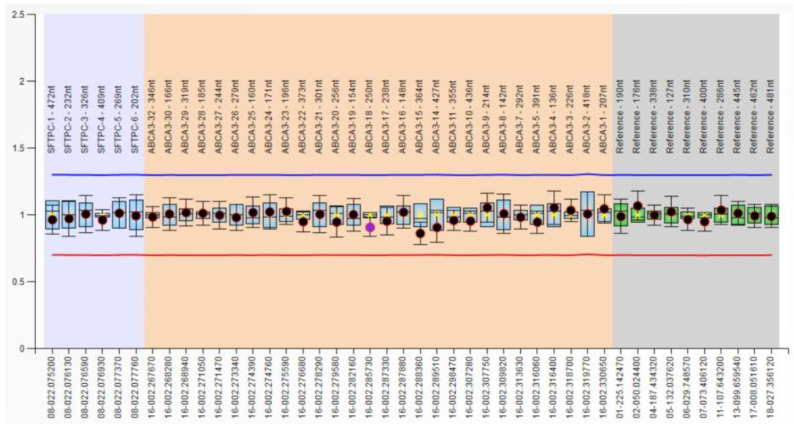
MLPA analysis. The blue line indicates the threshold for identifying a gain of genetic material (duplication), while the red line signifies the threshold for loss (deletion). Normal distribution of probes for P314-ABCA3-SFTPC Salsa MLPA probe mix.

Our MLPA results showed that the most frequent results were normal, with no CNV identified in these two genes, *ABCA3* and *SFTPC*, investigated in our patients. The dosage quotient of these probes was between 0.80 and 1.20, and the results are presented in Figure 1.

**Figure 2:**
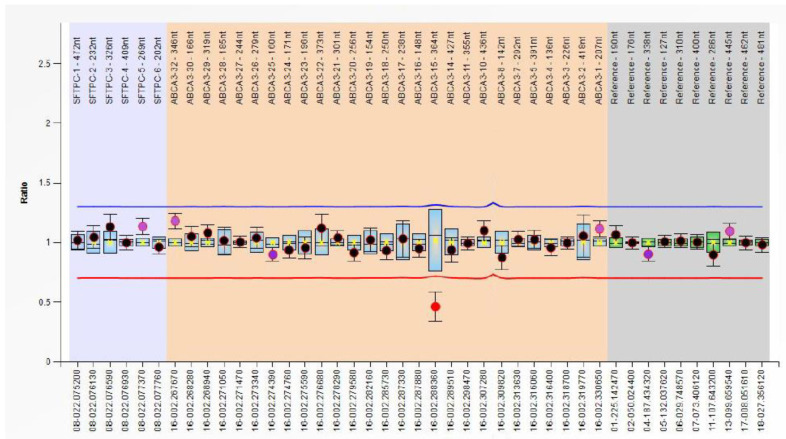
MLPA identification of a decreased signal in exon 15 of the ABCA3 gene. The blue line indicates the threshold for identifying a gain of genetic material (duplication), while the red line signifies the threshold for loss (deletion).

Abnormal results were identified in three cases, represented by abnormal signals in *ABCA3* gene exon 15 (Figure 2); the ratio of this signal was 0.46, with a normal ratio for the other exons. Two of these cases exhibit a severe manifestation of RDS, necessitating prolonged mechanical ventilation. In one of these cases, there was an additional complication of pulmonary hemorrhage, and the infant died four days after birth.

Also, exon 9 of gene *ABCA3* was identified as an abnormal reduced signal, with a ratio of 0.51. This patient necessitated extended early postnatal resuscitation. Subsequently, he developed a severe form of RDS, requiring mechanical ventilation for 238 h. Two administrations of surfactant were necessary. Throughout the treatment, there was an improvement in the general condition and hemodynamic status following the initiation of corticosteroid therapy, allowing for the possibility of discharge from the NICU at 25 days of life.

### 3.3. Genotype and Allele Frequency Analysis of ABCA3 SNPs and Their Effects on RDS Susceptibility

The investigated *ABCA3* SNPs were genotyped, and the distribution in RDS and control groups were analyzed according to codominant, dominant, recessive, and overdominant gene models. The results are represented in Table 2. The RDS and control groups were in Hardy–Weinburg equilibrium (HWE) (*p* < 0.05), except rs170447 (c.1741+33T>C) for the control group (*p* = 0.23).

The genotype distribution in these two groups (RDS and control) was similar, with a predominance of a heterozygous genotype for rs170447 (c.1741+33T>C) and the homozygous wild-type genotype for rs323043 and rs13332514 (Table 2). We did not identify a significant correlation for *ABCA3* rs170447 (*p* > 0.05); similarly, no significant differences were identified for *ABCA3* rs323043 (c.1755G>C) and *ABCA3* rs13332514 (c.1059G>A) in RDS and controls under gene model analysis (Table 2). Analysis of genotype distribution in recessive and overdominant gene models does not reveal a significant association between the control and RDS groups. Similar non-significant results were identified in allele frequency analysis (Table 3).

Furthermore, after adjusting for gestational age, treated as a categorical variable in the logistic regression model, no statistically significant association was observed for the examined SNPs.

Additionally, we applied a univariate analysis using the Kaplan–Meyer method, and we found no association between *ABCA3* rs170447 (c.1741+33T>C), rs323043 (c.1755G>C), and rs13332514 (c.1059G>A) variant genotypes and overall survival within the RDS group. Moreover, no correlation was observed between the presence of the specified genotype and the severity of the respiratory distress syndrome or outcomes in the case group.

### 3.4. Haplotype Analysis

Haplotype analysis of *ABCA3* rs170447 (c.1741+33T>C), rs323043 (c.1755G>C), and rs13332514 (c.1059G>A) was performed using Haplotype analysis v1.05 available at Georg-August-Universität Göttingen. Five haplotypes were analyzed with a frequency of >0.01 (TGG, CGG, CCG, CGA, and TGA). The reference haplotype was chosen after haplotype-based GLM regression, and the results establish that the C-G-G haplotype is the reference. The risk haplotype analysis revealed that *ABCA3* haplotypes are not significantly associated with increasing RDS risk.

Additionally, rs323043 (c.1755G>C) and rs170447 (c.1741+33T>C) were in linkage disequilibrium (D′ 0.97; r^2^ 0.21). For this analysis, we used the Linkage Disequilibrium Calculator from the Ensembl genome browser [35].

## 4. Discussion

Surfactant is a complex lipoprotein that significantly prevents alveolar collapse during exhalation, reducing the surface tension of air–liquid. This reduction in tension is vital for averting the collapse of alveoli during the exhalation phase. Pulmonary surfactant is synthesized by Type II alveolar cells, named pneumocytes, and is composed mainly of phospholipids (70–80%), protein (10%), and lipids (10%). The primary surface-active material from the structure is phospholipid dipalmitoylphosphatidylcholine (DPPC) and SP-A, SP-B, SP-C, and SP-D proteins [36]. The alveolar type II cells are differentiated from cuboidal epithelial cells, which develop cytoplasmatic lamellar bodies [37]. Surfactant production from glycogen is indicated by the presence of lamellar bodies, where it is stored [38].

Premature birth is the most common cause behind surfactant deficiency, often leading to a congenital deficit characterized by respiratory syndrome distress. Furthermore, variations in genes responsible for surfactant protein synthesis, namely *SFTPB*, *SFTPC*, and *ABCA3*, contribute to this congenital deficiency [39].

Next-generation sequencing (NGS) techniques have transformed the screening of genetic defects, playing a crucial role in advancing modern molecular medicine [40,41]. These methods excel in the precise detection of SNVs. However, a significant challenge in utilizing NGS for identifying SNVs in patients lies in accurately predicting or determining their functional and pathological consequences. On the other hand, SVs, which often have a more significant pathological impact than SNVs or INDELs, present a more intricate diagnostic challenge. Unfortunately, SVs may go undetected by NGS methods, and their full characterization is usually unattainable through other molecular techniques such as fluorescence in situ hybridization (FISH), array comparative genomic hybridization (CGH array), SNP array, or MLPA [4,14]. Hence, there is a considerable interest in enhancing the identification and characterization of SVs, especially those implicated in various diseases. While genetic databases prove highly beneficial for interpreting variants in well-established diseases, there is a lack of information for most pathogenic variants published in surfactant disorders.

According to the data extracted from the Human Gene Mutation Database (HGMD) for the *ABCA3* and *SFTPC* genes, the majority of defects leading to surfactant deficiency—specifically, 97.6% and 95.6%, respectively—are attributed to SNVs and INDELs [42,43]. Similar data have been reported in the genome Aggregation Database (gnomAD) v4.0, which incorporates 7274 variants within the *ABCA3* gene and 1263 variants within the *SFTPC* gene. HGMD Professional 2023.4 describes 391 *ABCA3* variants and 94 *SFTPC* variants that have been documented. These variants were identified by examining 58 and 40 articles, respectively, providing detailed information on *ABCA3* gene variations in 460 out of 1360 subjects included and *SFTPC* gene variations in 254 out of 3871 subjects. Moreover, approximately 2% and 1% of subjects, respectively, can be attributed to SVs, identified through MLPA targeting the thirty-three exons of the *ABCA3* gene and the eight exons of the *SFTPC* gene.

In the current study, we examined preterm infants with RDS using the MLPA analysis to assess its utility within this patient population. Another objective was to explore the association between *ABCA3* rs170447 (c.1741+33T>C), rs323043 (c.1755G>C), and rs13332514 (c.1059G>A) gene polymorphisms and susceptibility to RDS, as well as their impact on the overall survival of the patients enrolled in the study.

In our study, we did not identify *ABCA3* and *SFTPC* CNVs. The MLPA analysis used for CNVs analysis identified abnormalities of a single probe, targeting *ABCA3* exon 15 and exon 9. These abnormal single probe results for exon 15 were identified in three patients and for exon 9 in one patient. According to the manufacturer’s recommendation for the interpretation results, one single probe abnormality cannot be considered a deletion/duplication. We repeated DNA isolation and MLPA analysis for those cases, and the results were similar. There are some limitations of the technique used, and one of these is represented by small sequence changes even when they are localized more than 20 base pairs from the ligation site, and these determine a reduced probe signal (like in our cases). Sequencing analysis was recommended but was not performed; the parents’ financial situation did not allow this.

MLPA enables the simultaneous detection of specific CNVs across multiple human genes, which is crucial, particularly in cancer research, providing precise details about amplified or reduced copy numbers at specific genomic locations [44,45,46]. MLPA and array CGH have established benchmarks for CNV detection in genetic diagnostics [47,48]. MLPA’s evolving protocol extends its utility beyond CNV detection, being applied in various genetic variations and pathologies like spinal muscular atrophy and Duchenne muscular dystrophy [49,50,51,52]. This expanded use includes SNP genotyping, short tandem repeat analysis, expression profiling, and methylation status determination [53]. MLPA is recognized as comparable to FISH for identifying common aneuploidies, making it a rapid and reliable tool for prenatal diagnosis. Timely tests on amniotic fluid samples are recommended to determine the copy numbers of prevalent aneuploidies [54].

MLPA analysis of 32 Hispanic probands with classic cystic fibrosis revealed recurring deletions in four patients, constituting 12.5% and 11.1% of unidentified alleles. Three additional patients exhibited seemingly novel deletions, but subsequent sequencing unveiled unique nucleotide deletions, causing false-positive MLPA signals [55]. A study by Stuppia et al. underscores the importance of interpreting MLPA results, emphasizing that perceived single exon deletions may result from sequence variations affecting probe hybridization. Such variations could be pathogenic point mutations or non-impactful polymorphisms, necessitating independent verification for accurate confirmation [56].

These findings underscore the importance of meticulously designing MLPA probes to eliminate the influence of single-nucleotide polymorphisms in the boundary region. Additionally, validating MLPA results is essential to exclude potential artifacts. Nanopore sequencing, particularly with adaptive sampling, emerges as a promising approach for validation.

Considering the abovementioned considerations, we have outlined the constraints, utility, and advantages of employing the MLPA technique across various diseases. However, based on the findings of our study, we cannot assert the universal utility of this technique in all neonatal pathologies, including RDS. MLPA is not the primary investigative technique for patients with RDS, but it is useful when DNA CNVs frequently cause the disease and other sequencing techniques are unavailable. It is important to note that direct comparisons with other studies are challenging since no studies have analogous datasets to the best of our knowledge.

The second part of our study focused on evidence that highlights the involvement of *ABCA3* through the association between three SNPs of this gene (c.1741+33T>C, an intronic variant, and two synonymous variants, c.1755G>C and c.1059G>A) and susceptibility to RDS and overall survival in preterm infants.

The statistical analysis investigating *ABCA3* SNPs based on gene models (codominant, dominant, recessive, and overdominant) applied to our data showed that the investigated SNPs were not associated with overall survival or RDS susceptibility. Similar results are found in another study, where, in the analysis of five SNPs (including c.1755G>C, c.1741+33T>C), no significant deviation between newborns with and without RDS was found [26,28]. The prevalent synonymous *ABCA3* variant, rs13332514 (c.1059G>A), exhibits a notably higher frequency in the Chinese population (0.39 in HAPMAP) compared to cohorts of European (MAF = 0.098) and African descent (MAF = 0.087) in the United States. Although studies link this variant and another less common synonymous variant, rs323043 (c.1755G>C), to RDS in Finnish and Chinese premature infant cohorts, our investigation did not establish a similar association in preterm infants with RDS from Eastern Europe. Consequently, the precise contribution of these synonymous *ABCA3* variants to RDS remains uncertain and warrants further examination [26,27,29].

To the best of our knowledge, *ABCA3* rs13332514 (c.1059G>A) and rs170447 (c.1741+33T>C) were previously investigated in only one RDS case–control study on infants of Finnish ancestry [27]. In contrast, our findings contradict this previously documented case–control study, where one of the investigated haplotypes was overrepresented in very premature children with RDS. This overrepresentation was attributed to the *ABCA3* rs13332514 (c.1059G>A) SNP, demonstrating an increased frequency of minor alleles in individuals with RDS. Furthermore, the study reported a notable association of *ABCA3* rs13332514 (c.1059G>A) with chronic lung disease.

Moreover, a simultaneous analysis of these three SNPs (c.1755G>C, c.1741+33T>C, and c.1059G>A) was performed only on the Chinese population. Nevertheless, SNP rs13332514 (c.1059G>A) exhibited deviation from Hardy–Weinberg equilibrium and was consequently excluded from subsequent analyses [26]. In comparison with the previous case–control study, where the frequency of the minor allele at the coding *ABCA3* rs323043 (c.1755G>C) was significantly increased and may be a risk factor in premature infants with RDS, our results are similar.

The allele frequency was similar for all three investigated SNPs compared to the databases. According to gnomAD and the NCBI browser, the allele frequency for rs170447 (c.1741+33T>C) is reported in the European population as being approximately 50%, similar to our results, where the A allele was identified in 48% of cases. The rs323043 (c.1755G>C) variant C allele is reported in 20.6% according to gnomAD, and in our study, the frequency was 23.8% in the RDS group and 23.9% in the control group. Similar allele frequencies were identified between our groups and the database regarding rs13332514 (c.1059G>A).

Considering the substantial evidence in the literature highlighting the pivotal role of genetic mechanisms in the pathogenesis of most RDS cases and recognizing the limited number of studies specifically exploring these SNPs directly, numerous medical facilities have undertaken clinical exome sequencing to elucidate the genetic basis of RDS. This approach highlighted patients with severe respiratory distress at birth, progressing to respiratory failure that requires transplantation, who exhibit these variants, along with other associated mutations [57,58,59,60,61]. Moreover, the incongruence observed in our findings and other case–control studies may be attributed to variations in the study populations, particularly regarding the prevalence of the variant allele of rs13332514 (c.1059G>A). The existing literature characterizes the minor allele of this variant as a potential susceptibility factor for RDS [62].

Based on SIFT (Sorting Intolerant from Tolerant) and the input queries of our investigated SNPs, the rs323043 (c.1755G>C) and rs13332514 (c.1059G>A) SNPs may be considered as predictions tolerated with *p* > 0.05 [63].

Haplotype analysis is a valuable tool for uncovering potential associations within gene polymorphisms that may not be apparent when assessing individual SNPs. In our study, haplotype analysis of SNPs was outside the essential association and did not highlight any increased risk of developing RDS.

Despite conflicting findings, our results are supported by data available in databases, frequencies reported in these databases, and assessments from case–control studies or prediction analytics software. Our study is the first case–control study that simultaneously investigated rs323043 (c.1755G>C), rs170447 (c.1741+33T>C), and rs13332514 (c.1059G>A) and their haplotypes in association with RDS risk and overall survival. Moreover, we also investigated RDS patients using MLPA to analyze this technique’s utility as a first approach. However, our research does have certain constraints. One notable limitation is the relatively small sample size of the RDS group, which hinders a robust comparison between the control and RDS groups, and no adjacent data about the patient’s genome. Moreover, in understanding the positive association results, it is essential to consider the difference in gestational age between the two groups. Given the potential risk of RDS development linked to inadequate surfactant production in the study group, the significance of this factor becomes apparent. Therefore, it is imperative to conduct further large-scale studies to substantiate and validate our findings.

## 5. Conclusions

In conclusion, our study determined that the rs170447 (c.1741+33T>C) and rs13332514 (c.1059G>A) variants of *ABCA3* do not exhibit associations with susceptibility or survival outcomes in individuals with RDS. Additionally, MLPA analysis cannot be considered a useful technique in investigating patients with RDS, considering that we did not detect any deletions or duplications in the analyzed patients with RDS. Consequently, we recommend opting for a molecular technique, such as sequencing, for genetic investigation in patients with RDS.

## Figures and Tables

**Table 1:** Clinical and demographic characteristics of the patients and controls (without RDS).

Variables	RDS Patients	Controls (without RDS)	*p*-Values
Gestational age (weeks): mean ± SD	31 ± 3.72	32 ± 2.80	<0.015 *
Gender: n (%)			
Female	26 (34.2)	80 (37.4)	0.678 ^‡^
Male	50 (65.5)	134 (62.6)	
Birth weight (g): mean ± SD	1473.10 ± 504.51	1620.20 ± 310.5	<0.003 *
Preterm labor, n (%)	37 (48.6)	122 (57)	0.228 ^‡^
Singleton pregnancy, n (%)	70 (92.1)	202 (94.4)	0.579 ^‡^
Antenatal care, n (%)	39 (51.3)	139 (64.9)	0.040 ^‡^
Mother’s age, mean ± SD	28.7 ± 3.65	29.5 ± 3.35	0.081 *
Antenatal steroids, n (%)	37 (48.6)	12 (5.6)	<0.001 ^‡^
PROM > 18 h, n (%)	23 (30.2)	32 (14.9)	<0.001 ^‡^
Chorioamnionitis, n (%)	10 (13.1)	16 (7.5)	0.160 ^‡^
Delivery mode, n (%)			
Spontaneous	26 (34.2)	152 (71)	<0.001 ^‡^
C-section	50 (65.8)	54 (11.6)	<0.001 ^‡^
Delivery in a tertiary center, n (%)	60 (78.9)	209 (94.8)	<0.001 ^‡^
Apgar Score, mean			
1 min	6	7	<0.001 *
5 min	7	8	0.079 *
Surfactant use, n (%)	66 (86.8)	-	-
LISA	19 (28.7)	-	-
INSURE	19 (28.7)	-	-
Standard	28 (42.4)	-	-
Need for subsequent surfactant doses, n (%)	7 (9.2)	-	-
Duration of non-invasive ventilation, hours (mean)	222.48 ± 180.2	25 ± 18.9	<0.001 *
Duration of MV, hours (mean)	210.3 ± 50.2	-	-
Chronic lung disease, n (%)	15 (19.7)	-	-
PDA: n (%)	10 (13.1)	4 (1.86)	<0.001 ^§^
Pulmonary hemorrhage, n (%)	4 (5.2)	-	-
NICU days, mean ± SD	27.4 ± 4.9	7 ± 4.9	<0.001 *
Deaths, n (%)	7 (9.2)	-	-

Note: n—number; PROM—premature rupture of membranes; LISA—less invasive surfactant administration; INSURE—intubation–surfactant administration–extubation; MV—mechanical ventilation; IVH—intraventricular hemorrhage; PDA—patent ductus arteriosus; NICU—neonatal intensive care unit; *p*-values were obtained from generalized linear models; significant results were considered when *p*-values < 0.05; * *t*-test; **^‡^** χ^2^-test; ^§^ χ^2^-test with Yates correction.

**Table 2:** Genotype distribution of investigated *ABCA3* SNPs (rs170447, rs323043, rs13332514).

*ABCA3* SNPs	Gene Models	Genotypes	Controls n (%)	RDS Cases n (%)	OR Crude (95%CI) *	*p*-Value
rs170447 (c.1741+33T>C)	Codominant	TT	44 (20.5)	15 (19.7)	Reference	-
		TC	139 (65)	49 (64.5)	1.03 (0.528–2.02)	1.00
		CC	31 (20.57)	12 (15.8)	1.13 (0.46–2.75)	0.82
	Dominant	TT	44 (19.75)	15 (19.7)	Reference	-
		TC + CC	170 (79.43)	61 (80.3)	1.05 (0.54–2.02)	1.00
rs323043 (c.1755G>C)	Codominant	GG	161 (75.2)	51 (67.1)	Reference	-
		GC	40 (18.7)	21 (26.9)	1.657 (0.89–3.06)	0.137
		CC	13 (6.1)	4 (6)	0.97 (0.30–3.11)	1.00
	Dominant	GG	161 (75.2)	51 (67.1)	Reference	-
		GC + CC	53 (24.8)	25 (32.9)	1.48 (0.84–2.63)	0.17
rs13332514 (c.1059G>A)	Codominant	GG	195 (91.1)	67 (85.9)	Reference	-
		GA	14 (6.54)	9 (14.5)	1.87 (0.77–4.52)	0.216
		AA	5 (2.36)	-	0.26 (0.01–4.82)	0.33
	Dominant	GG	195 (91.1)	67 (88.1)	Reference	-
		GA + AA	19 (8.9)	9 (11.9)	1.37 (0.59–3.19)	0.49

Note: OR—odds ratio; 95%CI—95% confidence interval; * unadjusted regression analysis; *p*-values obtained from generalized linear models with a binomial distribution. Significant results were considered when *p*-values < 0.05.

**Table 3:** Analysis of allele frequency and their associations with RDS.

*ABCA3* SNPs	Variant Alleles	Frequency of Variant Allele in Control Group (%)	Frequency of Variant Allele in RDS Group (%)	OR Crude (95% CI) *	*p*-Value
rs170447 (c.1741+33T>C)	C	46.9	48	1.04 (0.72–1.51)	0.85
rs323043 (c.1755G>C)	C	28.9	23.9	1.293 (0.79–2.09)	0.308
rs13332514 (c.1059G>A)	A	5.6	7	1.05 (0.48–2.33)	0.84

Note: 95% CI = 95% confidence interval; *—unadjusted odds ratio in the RDS and control group for allelic model; significant results were achieved when *p*-values < 0.05.

## Data Availability

Data are contained within the article.
